# Calculation of NNTs in RCTs with time-to-event outcomes: A literature review

**DOI:** 10.1186/1471-2288-9-21

**Published:** 2009-03-20

**Authors:** Mandy Hildebrandt, Elke Vervölgyi, Ralf Bender

**Affiliations:** 1 Institute for Quality and Efficiency in Health Care (IQWiG), Dillenburger Str. 27, 51105 Cologne, Germany; 2 Institute for Medical Biometry, Epidemiology and Informatics (IMBEI), Johannes-Gutenberg-University Mainz, 55101 Mainz, Germany; 3 Medical Faculty, University of Cologne, Joseph-Stelzmann-Str. 9, 50931 Cologne, Germany

## Abstract

**Background:**

The number needed to treat (NNT) is a well-known effect measure for reporting the results of clinical trials. In the case of time-to-event outcomes, the calculation of NNTs is more difficult than in the case of binary data. The frequency of using NNTs to report results of randomised controlled trials (RCT) investigating time-to-event outcomes and the adequacy of the applied calculation methods are unknown.

**Methods:**

We searched in PubMed for RCTs with parallel group design and individual randomisation, published in four frequently cited journals between 2003 and 2005. We evaluated the type of outcome, the frequency of reporting NNTs with corresponding confidence intervals, and assessed the adequacy of the methods used to calculate NNTs in the case of time-to-event outcomes.

**Results:**

The search resulted in 734 eligible RCTs. Of these, 373 RCTs investigated time-to-event outcomes and 361 analyzed binary data. In total, 62 articles reported NNTs (34 articles with time-to-event outcomes, 28 articles with binary outcomes). Of the 34 articles reporting NNTs derived from time-to-event outcomes, only 17 applied an appropriate calculation method. Of the 62 articles reporting NNTs, only 21 articles presented corresponding confidence intervals.

**Conclusion:**

The NNT is used as effect measure to present the results from RCTs with binary and time-to-event outcomes in the current medical literature. In the case of time-to-event data incorrect methods were frequently applied. Confidence intervals for NNTs were given in one third of the NNT reporting articles only. In summary, there is much room for improvement in the application of NNTs to present results of RCTs, especially where the outcome is time to an event.

## Background

The concept of the number needed to treat (NNT) was proposed by Laupacis et al. [1] in 1988 to provide clinicians with a useful measure of treatment benefit. It represents the average number of patients who must be treated to prevent one adverse outcome within a certain duration of follow-up time, and is calculated by inverting the absolute risk reduction (ARR) [1,2]. There is an intensive discussion about the comprehensibility and the usefulness of NNTs in the scientific literature [3-11]. The main mathematical arguments against the use of NNTs, namely undesirable distributional properties and that NNT is undefined if ARR = 0, are justified. However, mathematical arguments lose their importance when NNT is considered just as a way to translate research results to patients, not as a tool for statistical computations [3,12]. It is also questioned by several authors whether NNTs are intuitively meaningful and helpful for physicians and patients [7-10]. Nevertheless, in the past years, the number needed to treat has become a well-known effect measure and is conventionally applied in randomised controlled trials (RCTs) with a binary outcome where the duration of follow-up time is fixed and the time to event plays no role or is ignored [12]. In 2001, the explanatory document of the Consolidated Standards of Reporting Trials (CONSORT) statement noted that NNTs could be helpful in expressing results for both binary and survival time data [13].

In RCTs with a binary outcome the calculation of NNTs is based on simple proportions referring to the fixed duration of follow-up (i.e. rates from a 2 × 2 table) [1,2,12]. In the case of time-to-event outcomes, the calculation of the number needed to treat is more difficult because varying follow-up times and censoring have to be taken into account [12].

Two basic methods have been proposed to calculate the number needed to treat in this situation. Altman & Andersen [14] proposed to calculate NNTs for one or several fixed time points based on survival probabilities estimated by the Kaplan-Meier survival curve or the Cox regression model. Due to the dependency on time, ARRs and NNTs refer to specific time points. A time specific NNT(t) is interpreted as the average number of patients needed to be treated to observe one event-free patient more in the treatment group than in the control group at time point t.

A second method was proposed by Lubsen et al. [15] and Mayne et al. [16], independently of each other. In both papers, it was proposed to use the reciprocal of the hazard difference rather than the risk difference to estimate NNTs for time-to-event outcomes. An argument for using hazards was that a distinction has to be made between trials of acute conditions with treatments of a short fixed duration and trials of chronic diseases and continuous treatments [15]. It was argued that in the case of chronic diseases and continuous treatments the calculation of NNTs by inverting the hazard difference would be more appropriate because an expression in units of person-time is required [15]. However, the NNT is an effect measure to quantify the impact of a treatment in terms of patient numbers that have to be treated to avoid one event within a certain length of follow-up time. The reciprocal of the hazard difference results in the average number of patient years (instead of patients) needed to observe one event less in the treatment group than in the control group. However, this explanation is only valid in the case of a constant hazard difference, i.e. if the distribution of the survival times is given by the exponential distribution [16] or the linear hazard rate distribution [17]. For all other survival time distributions the hazard difference and its reciprocal are time dependent. Moreover, the hazard difference is only a valid approximation of the risk difference if event rates are low, for instance less than 5% [16,18]. In all other cases the use of hazards to calculate NNTs is misleading. Therefore, in this paper the NNT is – as usual – considered as effect measure comparing the risks of two groups (treatment versus control) for a specific length of follow-up time in terms of patient numbers having to be treated to expect an avoided event in one patient.

Nuovo et al. [19] investigated the frequency of reporting NNTs in RCTs published in leading medical journals in the years 1989, 1992, 1995, and 1998. They found that only about 2% of eligible articles reported NNTs and concluded that this effect measure was underused in the medical literature.

The main objectives of our review are to investigate the frequency of reporting NNTs in RCTs published in leading medical journals in the years 2003–2005 and to assess whether the methods applied for their calculation were appropriate in the case of time-to-event outcomes. We also assessed whether confidence intervals were reported to describe the uncertainty of the estimated NNT measures for both time-to-event and binary outcomes.

## Methods

**Figure 1:**
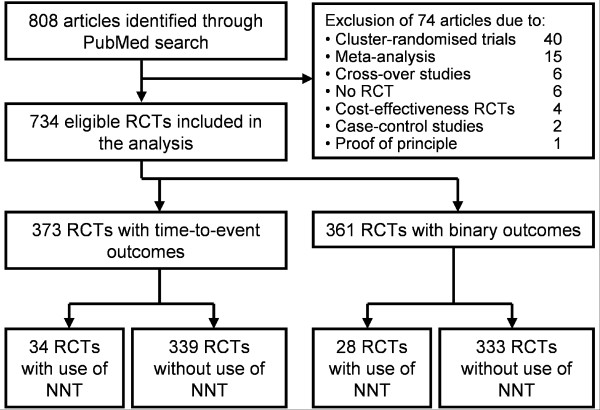
**Study selection procedure to identify randomised controlled trials (RCTs) reporting the number needed to treat (NNT) in leading medical journals in the years 2003–2005**.

Articles published in the years 2003 to 2005 in the following four frequently cited journals were evaluated: *BMJ, JAMA, New England Journal of Medicine (NEJM)*, and *Lancet*. The search was limited to articles with available abstracts and publication date 2003/01/01 to 2005/12/31. In a first step, each journal was searched using PubMed to identify articles reporting results of RCTs. Eligible articles included single studies that reported a parallel group design and an individual randomisation process; other articles were excluded (Figure 1). All titles and abstracts of the retrieved articles were screened to exclude obviously non-eligible articles. In a second step, the full texts of all eligible articles were then analysed to identify RCTs presenting NNTs (for any outcome) and RCTs investigating time-to-event outcomes. The articles were screened using the text search function. The terms used to identify the number needed to treat were "number", "need", "treat", and "NNT". The terms used to identify survival time data were: "survival", "Kaplan", "Cox", "life", and "time". If the screening results were negative or unclear, the methods sections of the articles were also reviewed manually to identify any use of NNTs and survival data.

We assessed, whether the methods used to calculate NNTs from time-to-event outcomes were appropriate. According to the methodology described in the literature [12,14-16,18] we considered a method as appropriate if the NNT was calculated either from survival probabilities estimated by means of the Kaplan-Meier method or the Cox regression model [14] or if it was calculated as the inverse of the hazard difference and both assumptions mentioned above are met (constant hazard difference and low event rates) [15,16,18]. When the method to calculate NNTs was not described in the article, we tried to verify the reported NNTs by recalculation from the presented data. The use of an appropriate method to calculate NNTs was possible if the corresponding Kaplan-Meier survival or incidence curves were presented. In this case we were able to recalculate the NNT as follows. At first we identified the point of time at which the NNT was estimated. If no time point was given we used the latest time point of the Kaplan-Meier graph. From this time point we draw a vertical line to the top of the graph so that the curves of the treatment arms were crossed. From these cross points we draw horizontal lines to the y-axis and read off the corresponding survival probabilities for the different treatment arms as accurate as possible. These probabilities were then used for NNT calculation. When it was clear that an inappropriate method was used either by statements given in the text or by comparing the presented with the recalculated NNT, the method was classified as "inappropriate", otherwise as "appropriate".

We also assessed whether confidence intervals for the number needed to treat were provided. If the numbers at risk were given together with the Kaplan-Meier curve or were inferable because of lost-to-follow-up information or a hazard ratio with confidence interval was presented we were able to calculate also a confidence interval for the recalculated NNT by using one of methods proposed by Altman & Andersen [14]. If numbers at risk were given but not exactly for the required time point we used the numbers at risk for the corresponding nearest time point.

Additionally, we investigated the reporting of absolute risk reduction with corresponding confidence interval. To characterise the studies we further evaluated the median sample size of the studies reporting NNTs and whether the outcome for which the NNT was calculated was a primary or secondary endpoint.

## Results

A total of 808 articles were initially retrieved in the PubMed search, of which 734 met the inclusion criteria. Figure 1 shows the flow chart of this literature review. Of the eligible articles, 62 (8.4%) reported a number needed to treat (Table 1). One article used the method proposed by Lubsen et al. [15] but described the results as "number of patient years of treatment to save one life" and not as "NNT" or "number of patients ...". Thus, this article was classified as non-NNT-reporting article. The 62 NNT-reporting articles had a median sample size of 553, ranging from 47 to 12639. Furthermore, 56 of 62 (90.3%) articles calculated the number needed to treat for the primary endpoint, 5 (8.1%) for primary and secondary endpoints, and 1 study (1.6%) calculated the number needed to treat only for a secondary outcome. The distribution of the 734 articles across the four considered journals BMJ, JAMA, Lancet, and NEJM was 90, 199, 190, and 255 (Table 1). As the results indicated no trend over the three years we do not show the results of the single years. NEJM published the largest number of RCTs but had the lowest use of NNTs (19 of 255 articles), whereas the BMJ with the least number of RCTs represented the journal with the highest use of NNTs (13 of 90 articles). The BMJ was the journal with the largest percentage of articles presenting confidence intervals for NNT estimates (7 of 13, 53.8%).

**Table 1:** Reporting of the number needed to treat (NNT) and corresponding 95% confidence interval (CI) in randomised controlled trials (RCTs) in leading medical journals in the years 2003–2005

	No. of articles		
	
Journal	RCTs	NNT reporting	CI for NNT
BMJ	90	13	7
JAMA	199	16	4
Lancet	190	14	4
NEJM	255	19	6

**Total**	**734**	**62 (8.4%)**	**21 (33.9%)**

Time-to-event outcomes were investigated in 373 (51%) articles; the other 361 articles used binary outcomes. In 3 articles, survival techniques as well as 2 × 2 tables were used for data analysis. The use of both methods was adequate in these articles because the follow-up time was equal for all patients and no censoring occurred. As NNTs were calculated on the basis of 2 × 2 tables these articles were classified as RCTs using binary outcomes. Of the 62 articles reporting NNTs, 34 articles presented time-to-event outcomes and 28 presented binary outcomes. Of the 34 NNT-reporting articles with time-to-event outcomes, only 17 (50%) applied an appropriate calculation method (Table 2). In all these articles, the NNT calculation was clearly based on estimated survival probabilities by means of the Kaplan-Meier survival curve or the Cox regression model or the reported NNT equalled our recalculated NNT. In the remaining 17 (50%) of the 34 NNT-reporting articles with time-to-event outcomes the calculation was seemingly based on naive proportions (rates from 2 × 2 tables). This approach neglects varying follow-up times and censoring and was therefore classified as inappropriate. If possible, we recalculated the NNT based upon estimated survival probabilities. In Table 3 the published and recalculated NNTs of the 17 articles with 95% confidence intervals (if recalculation was possible) and the corresponding absolute differences are summarized. A table providing some details (citation, experimental and control intervention, outcomes, sample size, follow-up time, published NNT, and corresponding 95% confidence interval) of the 34 NNT-reporting articles with time-to-event outcomes is given as Additional file 1.

**Table 2:** Reporting of the number needed to treat (NNT) and corresponding 95% confidence interval (CI) in randomised controlled trials (RCTs) with time-to-event outcomes in leading medical journals in the years 2003–2005

	No. of articles			
	
Journal	RCTs with time-to-event data	NNT	Appropriate NNT calculation	CI for NNT
BMJ	17	2	0	0
JAMA	89	9	4	2
Lancet	111	10	6	1
NEJM	156	13	7	3

**Total**	**373**	**34 (9.1%)**	**17 (50%)**	**6 (17.6%)**

**Table 3:** Reported and recalculated NNTs with 95% confidence intervals (CIs) from 17 studies using inappropriate methods to calculate NNTs for time-to-event data

No.	Reported NNT	Reported 95% CI	Recalculated NNT	Recalculated 95% CI	Absolute difference (reported NNT – recalculated NNT)
**1**	14	-	17.5	9.2 – 171.9	-3.5
**2**	23	-	18.2	11.1 – 49.6	+4.8
**3**	10	-	14.7	7.8 – 117.6	-4.7
**4**	40	-	57.1	NNTB 24.2 to ∞ to NNTH 156.6	-17.1
**5**	O1: 2.2 O2: 6.1	-	O1: 2.0 O2: 4.5	O1: 1.7 – 2.7 O2: 2.8 – 15	O1: +0.2 O2: +1.6
**6**	TP1: 5–6 TP2: 12	TP1: 3.6 – 11.1 TP2: 6.3 – 74.6	*	-	-
**7**	138	77 – 641	*	-	-
**8**	38	-	*	-	-
**9**	9	6 – 14	*	-	-
**10**	O1: 40 O2: 118	-	O1: 33.3 O2: 100.0	O1: 19.3 – 123 O2: NNTB 46.8 to ∞ to NNTH 741.3	O1: +6.7 O2: +18.0
**11**	7.5	4.8 – 14.7	7.1	4.5 – 16.9	+0.4
**12**	39	-	38.2	21.7 – 158.1	+0.8
**13**	4.3	-	4.5	2.6 – 17.9	-0.2
**14**	5	-	4.9	3.7 – 7.1	+0.1
**15**	30	-	*	-	-
**16**	"Slightly more than 6"	-	*	-	-
**17**	NNS = 352	-	325.7	185.4 – 1337.5	+26.3

* A recalculation of the NNT was not possible because no sufficient information about the required survival probabilities was presented.

CI = confidence interval; NNS = number needed to screen; NNTB = number needed to treat for one patient to benefit; NNTH = number needed to treat for one patient to be harmed; O = outcome; TP = time point

To explain the methods of our calculations we present one typical example. One study provided the information "The number needed to treat to prevent 1 cardiovascular event would be 40 patients with IGT over 3.3 years". Additionally, the naive proportions of patients experiencing an event were given as 32/686 in the placebo group and 15/682 in the intervention group. Obviously, the result of NNT = 40 is based upon these naive proportions, because 1/(32/686-15/682)≈1/0.025 = 40. However, due to varying follow-up times and censoring, the naive proportions represent no valid estimates of the corresponding risks at time point 3.3 years, which is only the mean follow-up time. An adequate approach to estimate the required risks for a specified time point is given by the Kaplan-Meier method.

We enlarged the Kaplan-Meier incidence curve given in the paper and determined the corresponding risk estimates at time point 1200 days visually as accurate as possible. We found the risk values 0.0410 and 0.0235 for the placebo and the intervention group, respectively. Thus, the recalculated NNT is given by 1/(0.0410 - 0.0235) = 1/0.0175 = 57.1 and the reported NNT of 40 is about 30% too low.

In the 62 NNT-reporting articles, corresponding confidence intervals were presented in 21 studies (6 of the 34 studies with time-to-event outcomes and 15 of the 28 studies with binary outcomes). Among the 62 NNT-reporting articles, 1 article used the term "number needed to screen" (NNS), 2 articles used the terminology "number needed to treat for one patient to benefit" (NNTB) and harm (NNTH), respectively, and 1 article used the term "number needed to harm" (NNH).

The absolute risk reduction was given in 33 (53.2%) of the 62 NNT-reporting articles (17 with time-to-event data and 16 with binary data), a corresponding confidence interval for the absolute risk reduction was given in 21 (63.6%) of 33 articles (7 with time-to-event data and 14 with binary data).

## Discussion

The number needed to treat is used as effect measure to present the results from randomised controlled trials with binary and time-to-event outcomes. We found that in the case of survival time data incorrect methods were frequently applied. As the explanatory document of the CONSORT statement [13] described the number needed to treat in addition to other effect measures (risk ratio or risk reduction) as helpful for expressing results of both binary and survival time data, appropriate methods are required for the calculation of NNTs also for the situation of time-to-event data. Our finding that 50% of the NNT-reporting articles with survival time data used inadequate calculation methods underlines the requirement to point out that special methods based on survival time techniques have to be used to calculate NNTs in this situation. This observed proportion probably underestimates the true proportion because we classified the method to calculate NNTs as "appropriate" if the method used was unclear and the reported NNT equalled the recalculated NNT from survival probabilities. It could be that in fact naive proportions have been used (i.e. an inappropriate method) but the result haphazardly equalled the correct result based upon survival probabilities. Thus, the true proportion of NNT-reporting articles with survival time data and inadequate calculation methods may be even higher than the observed proportion of 50%. As the considered journals represent the leading journals in medical research it can be expected that a broader review containing also medical journals of lower rank would lead to even a higher proportion of papers with inadequate NNT calculation.

In this paper we did not judge whether the application of NNTs was helpful or useful in the specific situation. For example, it was argued that in the case of chronic diseases and continuous treatments the calculation of NNTs by inverting the risk differences is not useful because the duration of treatment is not taken into account [15]. We agree that in the case of continuous treatments one should be careful if a cost-effectiveness analysis shall be made on the basis of NNTs. The treatment costs depend on the duration of treatment and this is shorter than the follow-up time for patients having an event before the end of the study. Thus, simple NNTs are insufficient for cost-effectiveness analyses in the case of chronic diseases and continuous treatments. If the duration of treatment is important, more complicated methods are required, e.g. survival techniques for time dependent covariates. These methods are not considered in this paper because the problem of treatment duration is independent from the type of outcome (binary or time-to-event data). If the treatment duration plays a role in the analysis, it has to be considered in addition to the effect measure used, regardless of whether the effect measure is the NNT or any other measure (risk difference, odds ratio, hazard ratio). In general it is highly subjective whether NNTs are useful or not. Therefore, we did not judge the usefulness of reported NNTs in the specific situation but considered the frequency of NNT applications in RCTs published in major medical journals in the years 2003 to 2005 and verified whether the applied calculation methods were technically appropriate in the case of time-to-event outcomes.

The error produced by using an inadequate method to calculate NNTs is unpredictable. In a number of cases, there was no substantial difference between adequately and inadequately calculated NNTs. For example, one trial with inappropriate NNT calculation presented a number needed to treat of 39 which is nearly the same as the correct result of 38.2 obtained by the appropriate method proposed by Altman & Andersen [14]. However, in another trial the published NNT of 23 is 26.4% too large (absolute difference: +4.8) compared with the correct result of 18.2. In another example the published NNT of 10 is 32% too small (absolute difference: -4.7) compared with the correct result of 14.7 (Table 3). It has been argued that clinicians should not be overly concerned about inaccuracies that may arise from estimating NNTs inadequately from naive proportions, especially when using data from large RCTs with high rates of follow-up [20]. We agree that in the case of equal censoring in the two groups the difference between adequately and inadequately calculated NNTs is negligible in practice. However, if the amount of censoring is quite different between the experimental and control group, relevant differences between adequately and inadequately calculated NNTs can be obtained. Moreover, confidence intervals for NNTs will be too narrow if censoring is not taken into account because the values used for the effective sample sizes are too large. This is demonstrated in Table 3 where the recalculated confidence interval covers the reported confidence interval completely. Unfortunately, there was only one study in which a confidence interval for NNT was reported and a recalculation of the confidence interval was possible. As the application of survival techniques is standard in the analysis of RCTs with varying follow-up times to account for censoring there is no reason to accept inaccurate point or interval estimates for NNTs due to neglecting censoring.

According to the CONSORT statement [21] confidence intervals should be reported for estimated effect measures to indicate the precision of the estimates. Due to the unusual scale of NNTs their confidence intervals are difficult to describe if the effect is not significant [22]. This may be one reason why confidence intervals for the number needed to treat were given in one third of the investigated articles only (time-to-event and binary data). Nevertheless, the methodology to calculate confidence intervals for NNTs is described and explained in the statistical as well as in the medical literature [12,22-27], so that the unusual scale of NNTs should be no argument to disregard the CONSORT statement.

## Conclusion

In summary, there is much room for improvement in the application of the number needed to treat to present results of randomised controlled trials, especially where the outcome is time to an event. To account for censoring survival time techniques have to be used to calculate the number needed to treat. The common standard to provide confidence intervals to indicate the uncertainty of estimated effect measures should also be applied to the number needed to treat. In general, it should be carefully considered whether the use of the number needed to treat is sensible in the specific context. If the number needed to treat is applied the use of correct calculation methods is required as well as the presentation of point and interval estimates.

## Abbreviations

ARR: absolute risk reduction; BMJ: British Medical Journal; CONSORT: Consolidated Standards of Reporting Trials Statement; JAMA: Journal of the American Medical Association; NEJM: New England Journal of Medicine; NNH: number needed to harm; NNS: number needed to screen; NNT: number needed to treat; NNTB: number needed to treat for one person to benefit; NNTH: number needed to treat for one person to be harmed; RCT: randomised controlled trial.

## Competing interests

The authors declare that they have no competing interests.

## Authors' contributions

MH and RB contributed to the concept and design of the manuscript. MH wrote the initial draft of the manuscript. All authors contributed to the analysis and interpretation of the data and did a critical revision of the manuscript for important intellectual content. Finally, all authors contributed to the manuscript preparation, read, and approved the final manuscript.

## Pre-publication history

The pre-publication history for this paper can be accessed here:

http://www.biomedcentral.com/1471-2288/9/21/prepub

## Supplementary Material

Additional file 1 **Characteristics of 34 randomised controlled trials (RCTs) reporting the number needed to treat (NNT) for time-to-event outcomes in leading medical journals in the years 2003–2005.** Additional file 1 is a table providing some details (citation, experimental and control intervention, outcomes, sample size, follow-up time, published NNT, and corresponding 95% confidence interval) of the 34 NNT-reporting articles with time-to-event outcomes. Click here for file

## References

[B1] Laupacis A Sackett DL Roberts RS An assessment of clinically useful measures of the consequences of treatment N Engl J Med 1988 318 1728 1733 3374545 10.1056/NEJM198806303182605

[B2] Cook RJ Sackett DL The number needed to treat: A clinically useful measure of treatment effect BMJ 1995 310 452 454 7873954 10.1136/bmj.310.6977.452 PMC2548824

[B3] Walter SD Choice of effect measure for epidemiological data J Clin Epidemiol 2000 53 931 939 10.1016/S0895-4356(00)00210-9 11004419

[B4] Hutton JL Number needed to treat: Properties and problems J R Stat Soc A 2000 163 403 415

[B5] Altman DG Deeks JJ Comments on the paper by Hutton J R Stat Soc A 2000 163 415 416

[B6] Lesaffre E Pledger G Comments on the paper by Hutton J R Stat Soc A 2000 163 417

[B7] Kristiansen IS Gyrd-Hansen D Nexøe J Nielsen JB Number needed to treat: Easily understood and intuitively meaningful? Theoretical considerations and a randomized trial J Clin Epidemiol 2002 55 888 892 10.1016/S0895-4356(02)00432-8 12393076

[B8] Grieve AP The number needed to treat: A useful clinical measure or a case of the Emperor's new clothes? Pharmaceut Stat 2003 2 87 102 10.1002/pst.33

[B9] Halvorsen PA Kristiansen IS Decisions on drug therapies by numbers needed to treat: A randomized trial Arch Intern Med 2005 165 1140 1146 10.1001/archinte.165.10.1140 15911727

[B10] Nexøe J Kristiansen IS Gyrd-Hansen D Nielsen JB Influence of number needed to treat, costs and outcome on preferences for a preventive drug Fam Pract 2005 22 126 131 10.1093/fampra/cmh706 15640298

[B11] Halvorsen PA Selmer R Kristiansen IS Different ways to describe the benefits of risk-reducing treatments: A randomized trial Ann Intern Med 2007 146 848 856 17577004 10.7326/0003-4819-146-12-200706190-00006

[B12] Bender R Armitage P, Colton T Number needed to treat (NNT) Encyclopedia of Biostatistics 2005 Chichester: Wiley 3752 3761

[B13] Altman DG Schulz KF Moher D Egger M Davidoff F Elbourne DR Gøtzsche PC Lang T for the CONSORT Group The revised CONSORT statement for reporting randomized trials: Explanation and elaboration Ann Intern Med 2001 134 663 694 11304107 10.7326/0003-4819-134-8-200104170-00012

[B14] Altman DG Andersen PK Calculating the number needed to treat where the outcome is time to an event BMJ 1999 319 1492 1495 10582940 10.1136/bmj.319.7223.1492 PMC1117211

[B15] Lubsen J Hoes A Grobbee D Implications of trial results: The potentially misleading notations of number needed to treat and average duration life gained Lancet 2000 356 1757 1759 10.1016/S0140-6736(00)03215-3 11095272

[B16] Mayne TJ Whalen E Vu A Annualized was found better than absolute risk reduction in the calculation of number needed to treat in chronic conditions J Clin Epidemiol 2006 59 217 223 10.1016/j.jclinepi.2005.07.006 16488351

[B17] Lin C-T Wu SJS Balakrishnan N Parameter estimation for the linear hazard rate distribution based on records and inter-record times Commun Stat A 2003 32 729 748

[B18] Liu GF Wang J Liu K Snavely DB Confidence intervals for an exposure adjusted incidence rate difference with applications to clinical trials Stat Med 2006 25 1275 1286 10.1002/sim.2335 16138360

[B19] Nuovo J Melnikow J Chang D Reporting number needed to treat and absolute risk reduction in randomized controlled trials JAMA 2002 287 2813 2814 10.1001/jama.287.21.2813 12038920

[B20] de Lemos ML NNT for studies with long-term follow-up (Letter) CMAJ 2005 172 613 author reply 613–615 15738471 10.1503/cmaj.1041426 PMC550612

[B21] Moher D Schulz KF Altman DG for the CONSORT Group The CONSORT statement: Revised recommendations for improving the quality of reports of parallel-group randomized trials Ann Intern Med 2001 134 657 662 11304106 10.7326/0003-4819-134-8-200104170-00011

[B22] Altman DG Confidence intervals for the number needed to treat BMJ 1998 317 1309 1312 9804726 10.1136/bmj.317.7168.1309 PMC1114210

[B23] Lesaffre E Pledger G A note on the number needed to treat Control Clin Trials 1999 20 439 447 10.1016/S0197-2456(99)00018-5 10503803

[B24] Bender R Calculating confidence intervals for the number needed to treat Control Clin Trials 2001 22 102 110 10.1016/S0197-2456(00)00134-3 11306148

[B25] Barrowman NJ Missing the point (estimate)? Confidence intervals for the number needed to treat CMAJ 2002 166 1676 1677 12126323 PMC116155

[B26] Hildebrandt M Bender R Gehrmann U Blettner M Calculating confidence intervals for impact numbers BMC Med Res Methodol 2006 6 32 16836748 10.1186/1471-2288-6-32 PMC1569862

[B27] Bender R Kuss O Hildebrandt M Gehrmann U Estimating adjusted NNT measures in logistic regression analysis Stat Med 2007 26 5586 5595 10.1002/sim.3061 17879268

