# Supplementary material for: Calculation of NNTs in RCTs with time-to-event outcomes: A literature review
Source: BMC Med Res Methodol. 2009 Mar 20;9:21. doi: 10.1186/1471-2288-9-21 (PMC2666755; doi:10.1186/1471-2288-9-21)
Supplement: Additional file 1 — Characteristics of 34 randomised controlled trials (RCTs) reporting the number needed to treat (NNT) for time-to-event outcomes in leading medical journals in the years 2003–2005. Additional file 1 is a table providing some details (citation, experimental and control intervention, outcomes, sample size, follow-up time, published NNT, and corresponding 95% confidence interval) of the 34 NNT-reporting articles with time-to-event outcomes. [file 1471-2288-9-21-S1.pdf]

No	Citation	Study name	Experimental intervention	Control/standard intervention	Outcome	Type	Sample size	Follow-up time	NNT	95% Confidence interval (CI)
**1**	Lancet 2003;362:782-789	EUROPA	perindopril	placebo	cardiovascular death, myocardial infarction, or cardiac arrest	primary	12218	mean 4.2 years	50	-
**2**	Lancet 2003;362:772-776	CHARM-alternative trial	candesartan	placebo	cardiovascular death or hospital admission	primary	2028	median 33.7 months	14	-
**3**	Lancet 2003;362:95-102	none	radiotherapy	no radiotherapy	incidence of ipsilateral invasive disease	primary	1030	median 52.6 months	36	-
**4**	Lancet 2003;362:767-771	CHARM-added trial	candesartan	placebo	cardiovascular death or hospital admission	primary	2548	median 3.4 years mean 3 years	23	-
**5**	Lancet 2003;361:983-988	none	clindamycin	placebo	spontaneous preterm delivery or miscarriage	primary	494	cut-off gestational age: 37 weeks	10	§
**6**	JAMA 2003;290:486-494	STOP-NIDDM	acarbose	placebo	major cardiovascular events	primary	1429	mean 3.3 years (SD 1.2)	40	-
**7**	BMJ 2003;326:1367-	none	fluticasone propionat cream	base cream	time to relaps	primary	295	16 weeks	2.2	§
			fluticasone propionate ointment	base ointment					6.1	
**8**	NEJM 2003;349:733-742	none	angioplasty (referral hospitals)	accelerated treatment with intravenous alteplase (referral hospitals)	death, clinical evidence for reinfarction, or disabling stroke	primary	1572	30 days	17	-
			angioplasty (invasive-treatment center)	accelerated treatment with intravenous alteplase (invasive-treatment center)					18	-
**9**	NEJM 2003;348:383-393	none	intensive treatment	conventional treatment	cardiovascular events	primary	160	mean 7.8 years	5	-
**10**	NEJM 2003;349:2387-2398	none	doxazosin	placebo	overall clinical progression	primary	3047	mean 4.5 years	13.7	-
			finasteride						15.0	-
			combination therapy						8.4	-
**11**	NEJM 2003;348:2379-2385	none	17 alpha-hydroxyprogesterone caproate (17P)	placebo	preterm delivery before 37 weeks of gestation	primary	463	cut-off gestational age: 37 weeks	5-6	3.6 to 11.1
					preterm delivery before 32 weeks of gestation				12	6.3 to 74.6
**12**	NEJM 2003;348:1309-1321	none	eplerenone (in addition to optimal medical therapy)	placebo (in addition to optimal medical therapy)	time to death from any cause	primary	6632	mean 16 months	50	-
					time to death from cardiovascular causes or first hospitalization for a cardiovascular event				33	-
**13**	NEJM 2003;348:1425-1434	none	low-intensity warfarin	placebo	recurrent venous thromboembolism	primary	508	median 6.5 months	10	-
**14**	NEJM 2003;348:583-592	none	ACE-inhibitor	diuretic	cardiovascular events or death from any cause (all)	primary	6083	median 4.1 years	32	-
					cardiovascular events or death from any cause (men)				23	-

No	Citation	Study name	Experimental intervention	Control/standard intervention	Outcome	Type	Sample size	Follow-up time	NNT	95% Confidence interval (CI)
**15**	JAMA 2004;292:1307-1316	Phase Z of the A to Z trial	simvastatin	placebo	cardiovascular death	primary	4497	median 24 months (range 6 to 24)	77	-
					new-onset congestive heart failure				77	-
**16**	JAMA 2004;292:2217-2226	CAMELOT	amlodipine	placebo	cardiovascular event	primary	1318	2 years	16	-
**17**	Lancet 2004;364:685-696	CARDS	atorvastatin	placebo	time to first coronary heart disease events, coronary revascularisation, or stroke	primary	2841	median 3.9 years	27	-
**18**	Lancet 2004;363:594-599	none	autologous renal tumour cell vaccine	no adjuvant treatment	tumour progression or death	primary	553	70 months	8	-
**19**	Lancet;363:1757-1763	B-Aware	BIS-guided anaesthesia	routine care	confirmed awareness	primary	2503	30 days	138	77 to 641
**20**	NEJM 2004;350:11-20	none	valacyclovir	placebo	HSV2-infection	primary	1484	1 year	38	-
**21**	NEJM 2004;351:1197-1205	none	prophylactic radiofrequency catheter ablation of accessory pathways	no ablation	arrhythmic events	primary	47	median 34 months	2	1.4 to 3.1
**22**	NEJM 2004;350:459-468	none	strontium ranelate	placebo	vertebral fractures	primary	1649	3 years	9	6 to 14
**23**	JAMA 2005;294:211-217	none	intervention	usual care	hospice referral rates	pr.+sec.	206	30 days	5	-
**24**	JAMA 2005;294:1794-1798	CHARM	candesartan (in addition to optimal therapy)	placebo (in addition to optimal therapy)	cardiovascular death or nonfatal myocardial infarction	pr.+sec.	7599	median 37.7 months	40	-
					nonfatal myocardial infarction				118	-
**25**	JAMA 2005;294:3093-3100	PAPABEAR	amiodarone	placebo	atrial tachyarrhythmias during first 6 days	primary	601	1 year	7.5	4.8 to 14.7 (§)
**26**	JAMA 2005;294:1224-1232	PCI_CLARITY	clopidogrel	placebo	cardiovascular death, recurrent myocardial infarction, or stroke after PCI	pr.+sec.	1863	30 days	39	-
**27**	JAMA 2005;293:1082-1088	none	folate + mecobalamin	placebo	hip fractures	pr.+sec.	628	2 years	14	9 to 28 (§)
**28**	JAMA 2005;293:2601-2608	none	complicated grief treatment	interpersonal psychotherapy	treatment response	primary	102	19 weeks	4.3	-
**29**	Lancet 2005;365:2007-2013	none	immediate treatment with antiepileptic drug	deferred treatment with antiepileptic drug	recurrent seizure after first seizure	primary	1443	2 years	14	-
					recurrent seizure after multiple seizures at randomization				5	-
**30**	Lancet 2005;366:37-43	none	chloramphenicol eye drops	placebo eye drops	clinical cure at day 7	primary	326	7 days	14	§
**31**	NEJM 2005;353:977-987	none	levofloxacin	placebo	febrile neutropenia	primary	760	60 days	5	§
**32**	NEJM 2005;352:1425-1435	none	atorvastatin 10mg	atorvastatin 80mg	major cardiovascular event	primary	10001	median 4.9 years	30	-
**33**	NEJM 2005;352:777-785	none	recombinant activated factor VII (rFVIIa)	placebo	death or severe disability at 3 months	secondary	399	90 days	6.3	§
**34**	BMJ 2005;330:750-	none	screening by abdominal ultrasonography	no screening	number of deaths due to abdominal aortic aneurysm	primary	12639	mean 52 months	352*	-

Legend: § CI for risk difference given
* Number needed to screen (NNS)
